# rs35301225 polymorphism in miR-34a promotes development of human colon cancer by deregulation of 3′UTR in E2F1 in Chinese population

**DOI:** 10.1186/s12935-017-0402-1

**Published:** 2017-03-09

**Authors:** Haiqiang Jiang, Fengyuan Ge, Beina Hu, Lamei Wu, Huijian Yang, Huiyun Wang

**Affiliations:** 1 Department of Clinical Laboratory, Jiangyin Hospital of Chinese Traditional Medicine, Wuxi, China; 2 Department of Clinical Laboratory, Anting Hospital, Shanghai, China; 3 Department of Clinical Laboratory, Central Hospital of Jiading District, Shanghai, China

**Keywords:** Genotype, miR-34a, Tumor growth, E2F1, SNP

## Abstract

**Background:**

Previous reports have revealed that down-regulation of miR-34a expression can promote colorectal cancer (CRC) cell growth by targeting cell cycle-related transcriptional factor E2F1. To date, the function of the single nucleotide polymorphism (SNP) located in the mature region of miR-34a has not been investigated.

**Methods:**

We performed a case–control study including 685 CRC patients and 618 cancer-free controls. Genotyping, real-time PCR assay, cell transfection, and the dual luciferase reporter assay were used in our study. Cell proliferation and cell cycle analysis were measured in CRC cells including Hct-116 and SW480. The overall survival of different genotypes was also investigated.

**Results:**

We found that the rs35301225 polymorphism in miR-34a was involved in the occurrence of CRC by acting as a tumor suppressor by down-regulation of tumor-promoting gene E2F1. C/A SNP of miR-34a could promote CRC cell proliferation by up-regulation of E2F1. Also, C/A genotype can change the cell cycle by increasing the S phase percentage. Moreover, the SNP in rs35301225 of miR-34a was associated with tumor size and tumor differentiation, as well as metastasis in CRC patients; C/A SNP was related to the significantly enhanced expression of E2F1 and shorter survival in post-surgery CRC patients.

**Conclusions:**

rs35301225 in miR-34a was highly associated with a decreased risk of CRC in a Chinese population and might serve as a novel biomarker for colon cancer.

## Background

Colorectal cancer (CRC) is cancer in the colon or rectum. Risk factors for CRC include lifestyle, older age, and inherited genetic disorders [4, 5]. CRC recurrence rates are high among all populations, and surgery and combination chemotherapies have been shown to confer only modest survival benefits in advanced CRC, resulting in an overall 5-year survival rate of 24% [1]. Therefore, the development of new strategies for its primary prevention, early diagnosis, metastasis inhibition, and treatments are urgently needed.

miRNAs are a class of small non-coding RNA molecules that regulate gene expression by binding to partially complementary recognition sequences of target mRNAs [2, 3]. miRNAs have important roles in various biological and pathological processes, such as development, cell proliferation, differentiation, apoptosis, inflammation, stress response and migration [4–6]. Single nucleotide polymorphism (SNP) of miRNA was a novel mechanistic explanation for different targeting gene expression, the variant in miRNA especially in the mature form of miRNA could switch the binding force of target site and thus to cause increased transcription of targeting genes [7, 8].

Early in 2011, Zauli et al. reported that miR-34a was down-regulated in human leukemia which was essential in the development of leukemia by targeting two oncogenic factors: E2F1 and B-myc [9]. E2F1 expression was affected in colon cancer patients [10–12]. However, there is no report concerning SNP of miR-34a. Therefore, in this study, we sought to assess the association between a candidate SNP in miR-34a and susceptibility to colon cancer and its precursor in a Chinese population group. We also investigated whether the risk-associated polymorphism contributes toward colon cancer patients outcome, and the detailed mechanism involved.

## Methods

### Clinical sample information

The Hospital-based case–control study consists of 685 patients newly diagnosed with CRC and 618 cancer-free controls. All the subjects were recruited from the Jiangyin Hospital of Chinese Traditional Medicine, Anting Hospital and Central Hospital of Jiading district between February 2009 and August 2015. Patients with other hematological disorders, previous history of cancers, radiotherapy and chemotherapy were excluded. The cancer-free control subjects from the same geographic area showed no evidence of a genetic relationship with the cases. The patients were classified according to World Health Organization classification. This study was approved by the Institutional Review Board of Jiangyin Hospital of Chinese Traditional Medicine, and every patient had written informed consent. The clinical features of all the cases and controls were presented in Table 1.

**Table 1:** Clinical characteristic of colon cancer patients and cancer-free controls

Variables	Cases (*n* = 685)		Controls (*n* = 618)		P*
	*N*	%	*N*	%	
Age (years)					
≤50	376	54.89	345	55.83	0.738
>50	309	45.11	273	44.17	
Gender					
Male	318	46.42	319	51.62	0.067
Female	367	53.58	299	48.38	
Tumor size (cm)					
≤5	478	69.78			
>5	207	30.22			
Differentiation					
Well	249	36.35			
Moderate	205	29.93			
Poor	231	33.72			
Metastasis					
Yes	412	60.15			
No	273	39.85			

* Two-sided Chi square test for either genotype distributions or allele frequencies between cases and controls

### Cell lines and cell culture

Colon cancer cell lines including Hct-116 and Sw-480 were purchased from American Type Culture Collection. All cells were cultured in Dulbecco modified Eagle medium (DMEM) purchased from Gibco (CA, USA) supplemented with 10% fetal bovine serum (Invitrogen, Carlsbad, USA) and grown in humidified 5% CO_2_ at 37 °C.

### Construction of plasmids

The total fragment of the E2F1 3′UTR and its pGL3 Luciferase Reporter Vectors mutated form were amplified. The PCR products were cloned into the pGL3 Luciferase Reporter Vectors (Promega, CA, USA). The construction containing both CC and AA type of miR-34a were also synthesized and cloned into pSilence 2.1-U6.

### Dual-luciferase reporter assay

The treated cells harvested 48 h after miRNA treatment, and the firefly luciferase expression was measured and normalized to Renilla activities. Dual-luciferase assays (Promega, Madison, WI) were performed according to the manufacturer’s protocol and detected with a Fluoroskan microplate reader (Thermo Labsystems, Helsinki, Finland). Transfection was repeated three times in triplicate.

### Cell proliferation assays

Cell proliferation was monitored using CCK-8 (Dojin Laboratories, Kumamoto, Japan) according to the manufacturer’s instructions. In short, the mock and infected cells were seeded at a density of 1 × 10^4^ cells/well in 96-well flat-bottom plates. CCK-8 was added to each well containing 100 μL of the culture medium, and the plate was incubated for 3 h at 37 °C. Viable cells were evaluated by measuring the absorbance at 450 nm, using a microplate reader.

### Immunohistochemistry (IHC)

Sections were stained according to the previous publication [11]. The section was incubated with in primary mouse anti-human Ab for E2F1(Ab112580), the sections were stained with DAB according to manufacturer’s protocols and mounted and photographed using a digitalized microscope camera (Nikon, Tokyo, Japan).

### Genotype

Genomic DNA was extracted from peripheral blood by using QIAamp DNA blood mini kits (Qiagen, Hilden, Germany) according to the manufacturer’s instructions. Genotyping was performed with the TaqMan SNP Genotyping Assay. The PCR reactions were carried out in a total volume of 5 μL containing TaqMan Universal Master Mix, SNP Genotyping AssayMix, DNase-free water and genomic DNA. The PCR conditions were 2 min at 50 °C, 10 min at 95 °C, followed by 40 cycles at 95 °C for 15 s and 60 °C for 1 min. The 384-well ABI 7900HT Real-Time PCR System was applied (ABI, CA, USA).

### Statistical analysis

All experiments were performed in triplicate and repeated at least three times. Data were expressed as mean ± SD. The association between rs35301225 genotypes and the risk of CRC was evaluated by calculating the odds ratios (ORs) and their 95% confidence intervals (CIs) using univariate and multivariate logistic regression analysis. Differences between two independent groups were tested with Student’s t test. All statistical analyses were carried out using SPSS version 18.0 and presented with Graphpad Prism software. Kaplan–Meier survival curves were plotted, and the log-rank test was done. The significance of various variables for survival was analyzed by the Cox proportional hazards model in a multivariate analysis. The results were considered to be statistically significant at P < 0.05.

## Results

### Clinical significance of rs35301225 in CRC

We first detected genotype frequencies in 685 CRC cases and 618 healthy controls, whose characteristics are listed in Table 1. As shown in Table 2, Chi square statistical analysis revealed that the genotypes of rs35301225 followed a Hardy–Weinberg equilibrium distribution pattern in the healthy control group (*P* = 0.51) (data not shown). Further statistical analysis demonstrated that the CA genotype and AA genotype presented a significantly increased risk of CRC (*P* < 0.0001; for CA: Odds ratio (OR) = 1.15, *P* < 0.0001 and for AA: OR = 3.71, *P* < 0.0001). Furthermore, the A carrier group also carried an increased risk of CRC (OR = 2.08, *P* < 0.0001). All ORs were adjusted for sex, age, smoking status, drinking history, and family cancer history.

**Table 2:** Genotype frequencies of the miR-34a rs35301225 in colon cancer patients and cancer-free controls

Genotype	Cases (*n* = 685)		Controls (*n* = 618)		OR (95%CI)^a^	*P* value^a^
	*N*	%	*N*	%		
rs35301225						
CC	189	27.59	328	53.07	1	*<0.0001*
CA	155	22.63	184	29.77	1.15 (1.02–1.11)	
AA	341	49.78	106	17.15	3.71 (1.12–1.21)	
A carrier	496	72.41	290	46.93	2.08 (1.19–1.29)	*<0.0001*

Italic values indicate statistically significant associations

^a^The ORs, 95% CIs and *P* value were calculated after adjusting for age, gender

### Stratified analysis of correlation between miR-34a polymorphism and CRC

Next, we conducted a stratified analysis to understand the correlation between the SNP *rs35301225* genotypes and the clinical characteristics of CRC (Table 3). We found a significant association of the rs35301225 genotypes with the tumor size, poor differentiation, and metastasis. A carrier was related to larger tumor and poor differentiation (*P* < 0.0001).

**Table 3:** Stratified analysis of rs35301225 genotype in clinical characteristic of gastric cancer patients

Feather	Genotype				CC vs. AA *P* value*	CC vs. A carrier *P* value*
	CC	CA	AA	A carrier		
Age (years)						
≤50	99	81	196	277	0.258	0.4399
>50	90	74	145	219		
Gender						
Male	80	69	169	238	0.11	0.1990
Female	109	86	172	258		
Differentiation grade						
Well	102	75	72	147	<0.0001	<0.0001
Moderate	48	69	74	143		
Poor	39	11	195	206		
Tumor size (cm)						
≤5	157	144	177	321	<0.0001	<0.0001
>5	32	11	164	175		
Metastasis						
Yes	147	134	131	265	<0.0001	
No	42	21	210	231		<0.0001

* Two-sided Chi square test for either genotype distributions or allele frequencies between cases and controls

### The effect of rs35301225 on the regulatory role of miR-34a on E2F1 expression

**Fig. 1:**
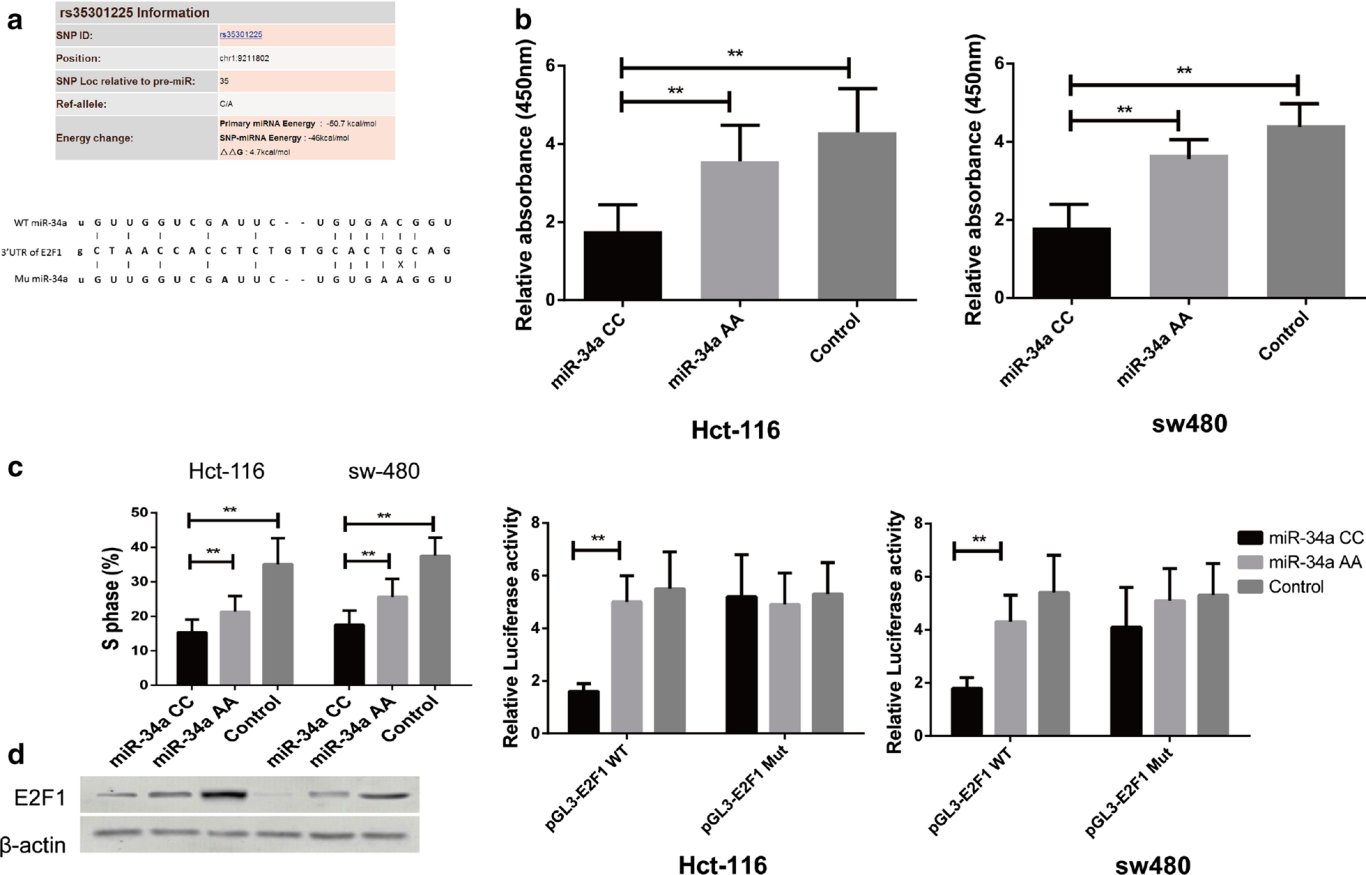
rs35301225 in miR-34a on the regulatory on E2F1 expression and cell proliferation. **a** Cell proliferation was measured by CCK8 assay in Hct-116 and sw-480 cell lines. Cells were treated with miR-34a harboring CC or AA genotype and control by vector transfection. **b** S phase of Hct-116 and sw-480 treated with miR-34a harboring CC or AA genotype was determined by flow cytometry. **c** Expression of E2F1 in of Hct-116 and sw-480 treated with miR-34a harboring CC or AA genotype were determined by western-blot. **d** Cells were co-transfected with miR-34a with CC or AA genotype, Renilla luciferase vector pGL3-Luc for 48 h. Both firefly and Renilla luciferase activities were measured in the same sample. Firefly luciferase signals were normalized with Renilla luciferase signals. Data was presented as the mean ± SEM. *P < 0.05 and **P < 0.01

Since the SNP rs35301225 was predicted to be located in the binding site of miR-34a on 3′UTR of E2F1, and the previous study revealed that the expression of E2F1 was regulated by miR-34a [9], we proposed that SNP of miR-34a might affect the regulation of E2F1 by miR-34a. We investigated the possible SNPs of miR-34a, and determined that there is an SNP in the mature form of miR-34, possibly affecting its binding on 3′UTR of E2F1 (Fig. 1a). To test whether or not the inhibitory effect of miR-34a was impacted by this SNP, we first measured cell proliferation by treated cells with miR-34a harboring different genotypes, including CC and AA. We found that cell proliferation could be suppressed by transfection of miR-34a including AA and CC compared to wild type control, but the proliferation of CRC cells transfected by miR-34a AA was significantly faster than miR-34a CC (Fig. 1b). Next, the cell cycle was analyzed: miR-34a AA can significantly increase the percentage of S phase of both Hct-116 and sw-480 cells compared to the CC genotype (Fig. 1c). Furthermore, the expression of E2F1 was analyzed by western-blot which indicated that E2F1 expression was significantly decreased in the CC group compared to AA (Fig. 1d). Next, we constructed pGL3 vectors containing the 3′UTR region of E2F1 and then co-transfected it with miR-34a with different genotypes in CRC cell lines. As shown in Fig. 1e, we found that the over-expression of miR-34a with the AA genotype could attenuate the suppression caused by miR-34a with CC in both colon cancer cell lines.

### C/A SNP was associated with high expression of E2F1 and shorter post-operative survival in clinical CRC patients

**Fig. 2:**
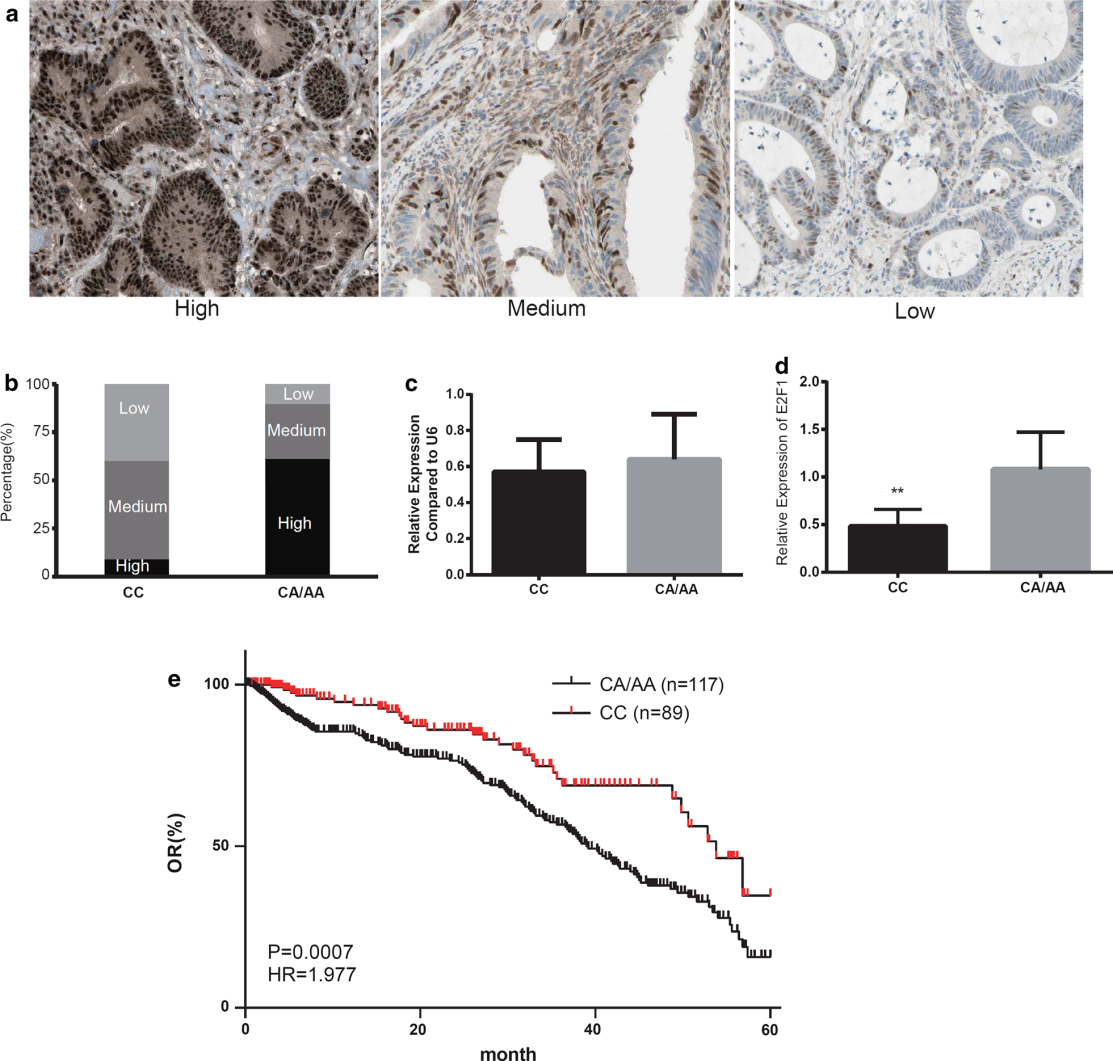
rs35301225 in miR-34a on the expression of E2F1 in clinical samples and overall survival of CC patients. **a** Representative figures for IHC staining of E2F1 in CC tumor section. **b** Comparison of components of IHC staining in both in CC and CA/AA genotype CC patients. **c**, **d** The expression level of E2F1 and miR-34a were determined by real-time PCR in CC and CA/AA genotype CC patients. **e** overall survival rate of post-surgery CC patients were analyzed by Kaplan–Meier survival curves. Data was presented as the mean ± SEM. *P < 0.05 and **P < 0.01

We also measured the expression of E2F1 in clinical samples with different genotypes of rs35301225. E2F1 expression was detected in human colon cancer by IHC. The staining was divided into high, medium, and low component categories. The CC group components were significantly different to the A carrier (CA/AA) groups in E2F1 expression (high 8.1%, medium 42.8%, and low 49.1% for the CC group; high 61.6%, medium 24.3%, and low 14.1% for the CA/AA groups, *P* < 0.001) (Fig. 2a, b). Real-time PCR further confirmed differences in E2F1 transcription, but there was no significant difference in miR-34a expression between these two groups (Fig. 2c, d). All of the results above might indicate that the A genotype in miR-34a might serve as a protective factor in CRC by affecting the binding of miR-34a on E2F1.

Among the total 685 CRC patients, we have 206 patients with follow-up survival data, and these can be further divided into CC (*n* = 89) and CA/AA (*n* = 117). We assessed the 5-year survival rate in the four groups. The 5-year survival rate in the CC group was 36.2%, which was significantly higher than in the CA/AA group with a survival rate of only 9.35% (95% CI: HR = 1.977, *P* = 0.008) (Fig. 2e).

## Discussion

Emerging data have shown that the aberrant expression of miRNAs contributes to tumorigenesis by inhibiting the expression of their target genes and that they potentially serve as biomarkers for prediction and prognosis in various cancers, including cervical cancer [13–15]. It has been identified that the SNP located in miRNA might also cause an abnormal function of the miRNA in regulating the potential target genes. For example, researchers have found that a genetic variant in miR-27a contributes to colon cancer susceptibility through affecting miR-27a and target gene expression [16]. SNPs occurring in miRNA sequences can affect processing and binding ability of mature miRNAs. Functional SNPs of miRNA-146a [19] have been found associated with cancer susceptibility, including lung cancer. Our results comprise new supplementary data for a function of a miRNA SNP contributing to colon cancer development. We found that the SNP of miR-34a has a C/A shift which can lead to dysregulation of the target gene E2F1, and cause relatively high expression of E2F1. Moreover, this SNP is clinically associated with bigger tumor size, lower differentiation, and tumor metastasis.

miR-34a is a tumor-suppressive miRNA which was reported to be capable of suppressing proliferation of a colon cell line by targeting E2F1 [17]. And with the accumulating of research data concerning miR-34a, more target of miR-34a were verified, Akao et al. reported that miR-34a was related to chemotherapy-resistant via suppressing of Sirt 1 and E2F3 also in human colon cancer [18]. Also, more and more study indicated miR-34a as a valuable index in colon cancer diagnosis. Recently, one of the group reported that circulating miR-34a capable of distinguishing patient groups with different diseases of the colon, and moreover, patients with advanced cancer from benign disease groups [19]. In present study, we also confirmed the regulation effect of miR-34a on E2F1 in human colon cancer, and furthermore, we find its regulation effect can be affected by SNP, CA/AA genotype of miR-34a was associated with stronger expression of E2F1 and relatively shorter post-operation survival, this result could be another explanation of variation expression of E2F1 within human colon cancer.

## Conclusions

We report the first evidence that the SNP rs35301225 located in miR-34a might be a protective factor to prevent the binding on 3′UTR of E2F1, which might suppress tumor growth in human CRC.

## Abbreviations

CRC: colorectal cancer
SNP: single-nucleotide polymorphism
3′UTR: three prime untranslated region
95% CI: 95 confidence interval
HR: hazard ratio
OD: odds Ratio
S. CC: CC genotype in rs35301225
AA: AA genotype in rs35301225
C/A: SNP C to A

## References

[1] Uthman OA, Jadidi E, Moradi T (2013). Socioeconomic position and incidence of gastric cancer: a systematic review and meta-analysis. J Epidemiol Community Health.

[2] Polytarchou C, Hommes DW, Palumbo T, Hatziapostolou M, Koutsioumpa M, Koukos G, van der Meulen-de Jong AE, Oikonomopoulos A, van Deen WK, Vorvis C (2015). MicroRNA214 is associated with progression of ulcerative colitis, and inhibition reduces development of colitis and colitis-associated cancer in mice. Gastroenterology.

[3] Nie H, Li J, Yang XM, Cao QZ, Feng MX, Xue F, Wei L, Qin W, Gu J, Xia Q (2015). Mineralocorticoid receptor suppresses cancer progression and the Warburg effect by modulating the miR-338-3p-PKLR axis in hepatocellular carcinoma. Hepatology.

[4] Fang F, Chang RM, Yu L, Lei X, Xiao S, Yang H, Yang LY (2015). MicroRNA-188-5p suppresses tumor cell proliferation and metastasis by directly targeting FGF5 in hepatocellular carcinoma. J Hepatol.

[5] Belgardt BF, Ahmed K, Spranger M, Latreille M, Denzler R, Kondratiuk N, von Meyenn F, Villena FN, Herrmanns K, Bosco D (2015). The microRNA-200 family regulates pancreatic beta cell survival in type 2 diabetes. Nat Med.

[6] Luna JM, Scheel TK, Danino T, Shaw KS, Mele A, Fak JJ, Nishiuchi E, Takacs CN, Catanese MT, de Jong YP (2015). Hepatitis C virus RNA functionally sequesters miR-122. Cell.

[7] Chen Z, Xu L, Ye X, Shen S, Li Z, Niu X, Lu S (2013). Polymorphisms of microRNA sequences or binding sites and lung cancer: a meta-analysis and systematic review. PLoS ONE.

[8] Mishra PJ, Mishra PJ, Banerjee D, Bertino JR (2008). MiRSNPs or MiR-polymorphisms, new players in microRNA mediated regulation of the cell: introducing microRNA pharmacogenomics. Cell Cycle.

[9] Zauli G, Voltan R, di Iasio MG, Bosco R, Melloni E, Sana ME, Secchiero P (2011). miR-34a induces the downregulation of both E2F1 and B-Myb oncogenes in leukemic cells. Clin Cancer Res.

[10] Bramis J, Zacharatos P, Papaconstantinou I, Kotsinas A, Sigala F, Korkolis DP, Nikiteas N, Pazaiti A, Kittas C, Bastounis E (2004). E2F-1 transcription factor immunoexpression is inversely associated with tumor growth in colon adenocarcinomas. Anticancer Res.

[11] Iwamoto M, Banerjee D, Menon LG, Jurkiewicz A, Rao PH, Kemeny NE, Fong Y, Jhanwar SC, Gorlick R, Bertino JR (2004). Overexpression of E2F-1 in lung and liver metastases of human colon cancer is associated with gene amplification. Cancer Biol Ther.

[12] Kitagawa M, Aonuma M, Lee SH, Fukutake S, McCormick F (2008). E2F-1 transcriptional activity is a critical determinant of Mdm2 antagonist-induced apoptosis in human tumor cell lines. Oncogene.

[13] Tang J, Zhuo H, Zhang X, Jiang R, Ji J, Deng L, Qian X, Zhang F, Sun B (2014). A novel biomarker Linc00974 interacting with KRT19 promotes proliferation and metastasis in hepatocellular carcinoma. Cell Death Dis.

[14] Schickel R, Boyerinas B, Park SM, Peter ME (2008). MicroRNAs: key players in the immune system, differentiation, tumorigenesis and cell death. Oncogene.

[15] Park H, Lee MJ, Jeong JY, Choi MC, Jung SG, Joo WD, Lee C, An HJ (2014). Dysregulated microRNA expression in adenocarcinoma of the uterine cervix: clinical impact of miR-363-3p. Gynecol Oncol.

[16] Sun Q, Gu H, Zeng Y, Xia Y, Wang Y, Jing Y, Yang L, Wang B (2010). Hsa-mir-27a genetic variant contributes to gastric cancer susceptibility through affecting miR-27a and target gene expression. Cancer Sci.

[17] Tazawa H, Tsuchiya N, Izumiya M, Nakagama H (2007). Tumor-suppressive miR-34a induces senescence-like growth arrest through modulation of the E2F pathway in human colon cancer cells. Proc Natl Acad Sci USA.

[18] Hiyoshi Y, Schetter AJ, Okayama H, Inamura K, Anami K, Nguyen GH, Horikawa I, Hawkes JE, Bowman ED, Leung SY (2015). Increased microRNA-34b and -34c predominantly expressed in stromal tissues is associated with poor prognosis in human colon cancer. PLoS ONE.

[19] Aherne ST, Madden SF, Hughes DJ, Pardini B, Naccarati A, Levy M, Vodicka P, Neary P, Dowling P, Clynes M (2015). Circulating miRNAs miR-34a and miR-150 associated with colorectal cancer progression. BMC Cancer.

